# Research on the risk spillover effect between China’s national carbon emissions trading market and crude oil futures market

**DOI:** 10.1371/journal.pone.0316353

**Published:** 2025-01-02

**Authors:** XueRong Bai, Yan Chen, Fan Yang

**Affiliations:** 1 School of Economics and Management, Nanjing Forestry University, Nanjing, China; 2 Academy of Chinese Ecological Progress and Forestry Development Studies, Nanjing Forestry University, Nanjing, China; 3 Jiangsu Industrial Technology Research Institute Application Technology Innovation Center, Nanjing, China; Guangdong University of Finance and Economics, CHINA

## Abstract

The development of China’s National Carbon Market has strengthened the inherent link between the carbon market and the broader energy market, providing a potential for cross-market risk transmission resonance. Studying the risk spillover effects between China’s National Carbon Market and the crude oil futures market is of significant practical importance, both in terms of carbon market development and carbon risk management. Based on the Maximal Overlap Discrete Wavelet Transform (MODWT), the price series are decomposed across multiple scales, and the risk spillover effects between the carbon market and the crude oil futures market are examined from both the time domain and the frequency domain. Methods such as wavelet energy decomposition, wavelet correlation, lead-lag analysis, and wavelet coherence are used to explore the mean spillover effects and volatility spillover effects (collectively referred to as risk spillover effects) across various scales. The study finds that China’s National Carbon Market exhibits a clear compliance-driven effect, with relatively low market liquidity. The crude oil futures market experiences frequent price fluctuations, primarily driven by long-term factors. In the time domain, the risk transmission resonance between the carbon market and the crude oil futures market is high, with significant positive correlations observed at the D1 to D4 scales, and noticeable mean spillover effects. In the frequency domain, at the D3 to D4 scales, the carbon market and the crude oil futures market exhibit similar volatility frequencies, indicating strong volatility spillover effects. Based on these findings, it is recommended that the trading volume of the carbon market be gradually increased to improve market liquidity. Furthermore, the risk monitoring and early warning mechanisms of China’s National Carbon Market should be improved. For carbon-emitting companies, enhancing awareness of carbon asset management and making informed investment and hedging decisions based on the correlation between the two markets is crucial.

## 1. Introduction

In recent years, the issue of global warming has attracted widespread attention from countries around the world and various sectors of society, becoming a focal point for many scholars. The World Meteorological Organization (WMO) released a report on the opening day of COP29, stating that from January to September 2024, the global average surface temperature was 1.54°C higher than the pre-industrial average. The Intergovernmental Panel on Climate Change (IPCC) Sixth Assessment Report indicates that, compared to the period 1850–1900, the global average surface temperature increased by 1.1°C during 2011–2020. It is projected that global greenhouse gas emissions could lead to a global temperature rise of over 1.5°C by 2030. Every incremental increase in global warming contributes to the occurrence of more frequent and severe impacts, making it urgent to curb global warming. Greenhouse gas emissions from the burning of fossil fuels are the primary cause of current global warming, and the traditional energy markets, along with energy-intensive industries, inevitably consume fossil fuels during their production processes [1]. Carbon markets can fully leverage market regulation and incentive mechanisms to drive high-carbon enterprises to reduce emissions in a cost-effective and efficient manner, balancing economic development with carbon emissions. Therefore, carbon markets have become an important tool for climate governance in most countries and a key policy instrument for China to achieve its "carbon peak and carbon neutrality" goals [2]. However, China’s National Carbon Market is still in its early stages of development, with the market not yet fully mature and significant issues of price volatility. This exposes the participating carbon-emitting enterprises to substantial risks. Furthermore, as the carbon market develops, its financial attributes become increasingly prominent [3], resulting in risk contagion between the carbon market and other financial markets. Compared to other markets, the energy market is more susceptible to the influence of the carbon market. Crude oil, as one of the main fossil fuels, has a tremendous impact on both the energy market and industrial development. As the carbon market experiences abnormal price fluctuations, the crude oil futures market also faces more severe challenges. Therefore, the practical goal of this paper is to deeply explore the risk spillover effects between the carbon market and the crude oil futures market in order to clarify the correlation characteristics between the two. Specifically, we will examine whether there is an interconnection between these two markets, whether it is positive or negative, whether there are lead-lag effects, and how their common frequency of fluctuations manifests. With these detailed research findings, we can further assess whether the carbon market is truly fulfilling its role in providing price signals, compare which market, the carbon market or the crude oil futures market, plays a more dominant role, and evaluate the current operational efficiency and market effectiveness of the carbon market. These findings provide important reference points for the government to improve the construction of the carbon market. The government can use these results to formulate and adjust related policies, aiming to optimize the operation mechanism of the carbon market, enhance market efficiency, and more effectively promote the development of a low-carbon economy. For most carbon-emitting enterprises, both the carbon market and the crude oil market are closely related to their production and operations. In order to operate more effectively, enterprises need to clearly understand the relationship between these two markets. Once they grasp this relationship, they will be able to better interpret market dynamics. With this understanding, enterprises can develop more timely and effective risk management strategies. These strategies will help them cope with potential market risks and ensure the stable development of the business. For investors, understanding the relationship between the carbon market and the crude oil futures market in the short, medium, and long terms will help them make more accurate investment decisions. Based on this understanding, investors can timely adjust their investment strategies, engage in risk avoidance, and capitalize on arbitrage opportunities to maximize investment returns. In conclusion, this study is of significant importance.

To date, a large number of scholars have studied the risk spillover effects between the carbon market and the crude oil futures market. Early research primarily focused on the European Union market. Most scholars believe that there is a close relationship between the EU carbon market and the oil market. Hammoudeh [4] argued that oil prices have a long-term asymmetric effect on carbon prices, and this effect is negative. Qiang Ji [5], Dhamija [6], and Uddin [7] also demonstrated, from different perspectives, that there is a close relationship between the oil market and the EU carbon market. Some scholars, however, argue that the effect of oil prices on carbon prices is relatively weak [8], and that coal prices have a greater influence [9]. Additionally, some studies suggest that there is no significant volatility spillover effect between the EU carbon market and the oil market [10]. Later, with the initiation of China’s carbon pilot markets, more and more scholars began to focus on the Chinese market. As there are multiple carbon pilot markets in China, the research objects vary among different scholars. Liu Zhiyang [11] found that crude oil futures have a significant positive effect on the weighted returns of China’s National Carbon Market. Cui, JX [12] argued that the oil market has a significant net spillover effect on the carbon market. Specifically, the Guangdong carbon market is positively correlated with the oil market, while the Shenzhen and Hubei carbon markets are negatively correlated with the oil market. Some scholars have also subdivided risk spillover effects, studying the mutual influence between different carbon markets and crude oil from various perspectives. Studies show that oil prices have both short-term adjustment effects and long-term persistent impacts on the Beijing, Shanghai, Guangdong, and Shenzhen carbon markets, with oil price fluctuations having a significant long-term and persistent effect on Hubei carbon prices [13]. Furthermore, some scholars argue that oil is not always the main influencing factor for the carbon market. For example, Zhao Lingdi [14] believes that the Shanghai carbon market has the closest linkage with the coal market, while the Beijing and Guangdong carbon markets are more closely related to the oil market. After the outbreak of two exogenous events, the COVID-19 pandemic and the Russia-Ukraine war, the level of global systemic risk spillover increased significantly. These two events had a worldwide impact, causing major economic losses and casualties. Additionally, they are considered key factors driving price changes in the carbon and energy markets [15]. Rizwan Ahmed [16] and Dong, Feng [17] found that during extreme events and crises (such as the COVID-19 pandemic and the Ukraine war), the risk spillover effect between the EU carbon market and the energy markets increased significantly. Similar changes were observed in China’s National Carbon Market and the U.S. carbon market as well [18]. In terms of research methods, most existing studies primarily use VAR models [19–21], GARCH-type [22–24] models, and DY index spillover models [25]. These models require certain assumptions regarding data stationarity. However, because carbon and crude oil futures prices often fail to meet stationarity requirements, the effectiveness of these models, the stability of their parameters, and the reliability of the conclusions may be compromised [26]. Some scholars have addressed this issue by using the Copula Quantile-Quantile regression method with EEMD to explore the risk spillover relationship between the two markets [27], thus avoiding the problems of non-stationarity and non-linearity in time series data. However, in terms of computational efficiency, statistical precision, reconstruction error, and handling boundary effects, the MODWT (Maximal Overlap Discrete Wavelet Transform, abbreviated as "MODWT") method outperforms others. In other words, the wavelet transform model still holds unparalleled advantages in dealing with non-stationary and nonlinear time series problems [28]. In addition to these advantages, wavelet transform is often referred to as a mathematical microscope [29]. Compared to traditional methods, MODWT allows for multi-scale decomposition, enabling data analysis from different dimensions. Furthermore, Zhang, YM [30] used TVP-VAR along with wavelet coherence to study the risk spillover effects between markets. The advantage of this model lies in its ability to explore market co-movements across scales and to study the dynamic changes in risk spillover effects. However, the TVP-VAR model introduces exogenous processes into both coefficients and volatility, which can lead to over-parameterization and the "curse of dimensionality."

In summary, significant achievements have been made in previous studies on the risk spillover between the carbon market and the crude oil futures market. These studies have not only explored the relationship between the two markets in depth but also revealed the complex dynamics between them through a variety of research methods, such as VAR models, GARCH models, and the DY index spillover model. Scholars have particularly demonstrated high levels of innovation in addressing issues related to the non-stationarity and non-linearity of time series data. For example, methods like the Copula quantile-quantile regression method with EEMD and wavelet transform models have effectively improved the accuracy and depth of the research. These findings have not only enriched the theoretical framework of financial market risk management but also provided important decision-making references for policymakers and market participants. However, despite the considerable progress made, several issues remain unresolved. On one hand, existing studies are often limited by the stationarity requirements of the data, which reduces the applicability of certain models in practical scenarios. On the other hand, there is a lack of in-depth discussion in the literature regarding the multi-scale risk spillover between China’s National Carbon Market and the crude oil futures market. This gap in the literature results in a limited understanding of the interaction between these two markets across different time scales. Moreover, as the largest carbon market in China and globally, the continuous development and improvement of China’s National Carbon Market is of paramount importance, both for China and for the world. Exploring the risk spillover effects between China’s National Carbon Market and the oil market is crucial for maintaining the stability of the carbon, energy, and financial markets. Additionally, few studies have comprehensively explored risk spillover from both mean spillover and volatility spillover perspectives from a multi-scale viewpoint. This limitation hinders a comprehensive understanding of the risk transmission between markets at different time scales. To address these issues, this paper selects the MODWT model, which has a significant advantage in handling non-stationary data. Moreover, the MODWT model excels in improving computational efficiency, ensuring statistical analysis accuracy, controlling reconstruction errors, and handling boundary effects. Based on this model, this paper uses a multi-scale decomposition framework, utilizing wavelet correlation and wavelet coherence methods to focus on exploring the risk spillover relationship between China’s National Carbon Market and the crude oil futures market from both mean spillover and volatility spillover perspectives. This study first helps deepen our understanding of the interactive relationship between the carbon market and the crude oil futures market, providing policymakers with scientific decision-making references. Secondly, it assists emission-control enterprises in avoiding risks rationally. It also provides effective market information for cross-market investors. Ultimately, this research contributes to China’s green, low-carbon, and sustainable development.

## 2. Methodology and data sources

### 2.1 Research framework

**Fig 1:**
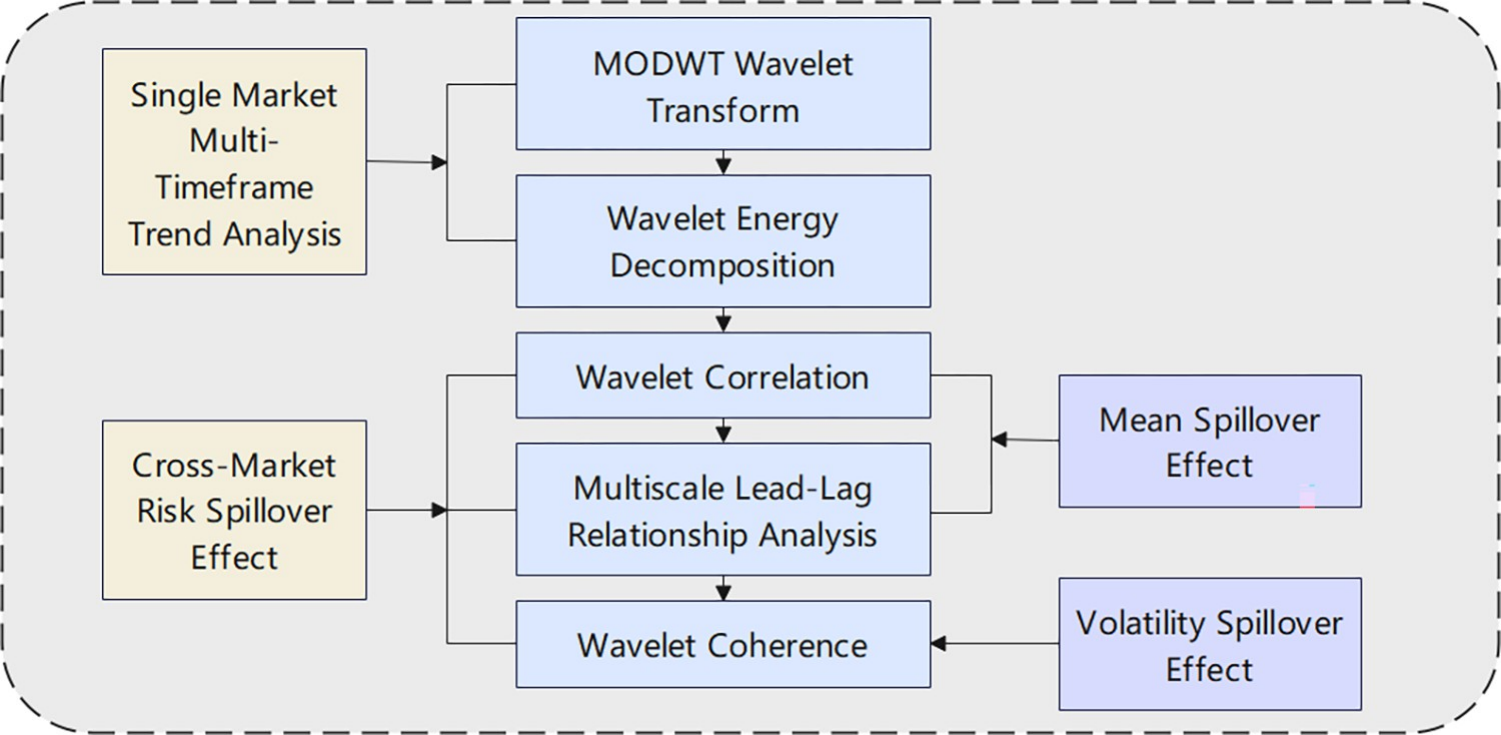
Diagram of the analytical framework.

The wavelet transform model has unparalleled advantages in handling non-stationary and nonlinear time series problems, making it highly compatible with the data used in this paper. Known as a mathematical microscope, the wavelet transform allows for multi-scale decomposition of data, enabling analysis from different scales. Based on this, this study primarily explores the risk spillover effects between the carbon market and the crude oil futures market from different scale perspectives. The risk spillover effects can be further subdivided into mean spillover effects and volatility spillover effects. The research is divided into the following five steps, as shown in Fig 1.

First, the maximum overlap discrete wavelet transform (MODWT) is applied to the log of carbon trading prices and the log of crude oil futures market prices. The volatility of the carbon market and the crude oil futures market at different scales is then examined from both the time domain and frequency domain perspectives.

Second, wavelet energy decomposition and multi-scale variance calculations are performed for the two markets to explore the proportion of short-term, medium-term, and long-term volatility in the overall volatility. This helps determine whether the volatility in the carbon market and the crude oil futures market is primarily driven by short-term or long-term fluctuations.

Third, based on the MODWT, the correlation coefficients and their confidence intervals between the two markets at different scales are calculated to investigate the correlation between the carbon market and the crude oil futures market at various scales. This is used to analyze the mean spillover relationship between the two markets.

Fourth, the lead-lag relationship between the carbon market and the crude oil futures market over the entire sample period is first analyzed, followed by an exploration of the lead-lag relationship between the two markets at multiple scales.

Fifth, wavelet coherence plots are generated to analyze the volatility spillover effects between the carbon market and the crude oil futures market in both the frequency and time domains.

### 2.2 Main research methods

#### 2.2.1 Maximal overlap discrete wavelet transform

MODWT is an extended form of the Discrete Wavelet Transform (DWT). By allowing greater overlap, it achieves higher time resolution and frequency characteristics in time series decomposition. MODWT is typically based on a specific pair of low-pass and high-pass filters.

The basic process of MODWT is as follows: (1) Filtering and downsampling: MODWT decomposes the signal through a series of filtering and downsampling operations. For each decomposition level, the signal passes through a pair of low-pass and high-pass filters, extracting approximation coefficients (low-frequency components) and detail coefficients (high-frequency components), respectively. (2) Overlap: Unlike traditional DWT, MODWT allows a greater degree of overlap, providing higher time and frequency resolution. The formula for MODWT is described as follows.


$$W_{jk}=\sum_{n=0}^{N-1}x[n]\cdot h_{j,k,n}$$

$$V_{jk}=\sum_{n=0}^{N-1}x[n]\cdot g_{j,k,n}$$

Of these, the *W*_*jk*_ and *V*_*jk*_ are the high-pass and low-pass coefficients at scale *j* and position *k*, the *h*_*j*,*k*,*n*_ and *g*_*j*,*k*,*n*_ are the coefficients of the corresponding high-pass and low-pass filters.

MODWT, as a form of wavelet transform that provides richer time-frequency information, can help analysts gain a more comprehensive understanding and analysis of time series data in the fields of economics and finance. It is commonly used for tasks such as signal denoising, feature extraction, volatility analysis, and time series forecasting.

#### 2.2.2 Wavelet correlation

Wavelet correlation analysis uses the wavelet transform to explore the correlation between different signals or time series. It can help identify the interrelationships between signals at different time scales, leading to a more comprehensive understanding of the signals’ properties. Given two signals *x* and *y*, their wavelet coefficients are *W*_*x*_ and *W*_*y*_, the wavelet correlation can be calculated using the following equation:

$$R_{xy}=\frac{\sum_{i=0}^{N-1}W_{x,i}\cdot W_{y,i}^{*}}{\sqrt{\sum_{i=0}^{N-1}{\left|W_{x,i}\right|}^{2}\cdot \sum_{i=0}^{N-1}{\left|W_{y,i}\right|}^{2}}}$$

Where denote the wavelet correlations of the signals x and y, and *W*_*x*,*y*_ and *W*_*y*,*i*_ are the wavelet coefficients of signals *x* and *y* at the *i* wavelet scale, respectively, and * denotes the complex conjugate.

#### 2.2.3 Lead-lag analysis

Lead-lag analysis in wavelet transform refers to the use of wavelet transform to explore the lead or lag relationship between different time series. Its mathematical formula can be described as given two time series *x[n]* and *y[n]* of length N, their correlation coefficients (*Rxy(*τ,*s)*.) at different scales s and time delays can be obtained through wavelet transforms τ. This correlation coefficient describes the correlation between two signals at a particular scale and delay period. Leading lag analysis is commonly used to determine whether one time series changes before or after another.

#### 2.2.4 Wavelet coherence

Wavelet coherence analysis is another method used to study the interrelationships between two time series [31]. It describes the phase coherence of a time series at different frequencies, thus revealing periodicity or resonance in the time series. Suppose there are two time series *x(t)* and *y(t)*, wavelet coherence analysis measures the coherence between them by calculating their wavelet correlation coefficients, *Rxy(s*,*t)*, at different scales, *s*, and times, *t*. The wavelet correlation coefficient is calculated as follows The formula for this correlation coefficient is:

$$R_{xy}(s,t)=\frac{{\left|W_{xy}(s,t)\right|}^{2}}{W_{xx}(s,t)\cdot W_{yy}(s,t)}$$

Where W_xy_(s, t) are the wavelet cross-spectra of *x(t)* and *y(t)*. *W*_*xx*_(*s*, *t*) and *W*_yy_(*s*, *t*) are the wavelet autospectra of *x(t)* and *y(t)*, respectively. The wavelet cross-spectrum represents the relationship between the frequency components of two signals at different scales and times.

In the field of economics and finance, wavelet coherence can help identify the correlation between market indicators or asset prices at different frequencies. Using wavelet coherence from a multiscale perspective is particularly useful for understanding the relationship between long-term trends and short-term fluctuations in financial markets.

### 2.3 Data sources and descriptive statistics

This study selects the daily closing prices of China’s National Carbon Market (CET) and the China Crude Oil Futures Market (OIL) as the research subjects. The sample period spans from July 16, 2021, to December 29, 2023, with all data denominated in Chinese yuan (CNY). After excluding non-common trading days, a total of 596 daily data points are retained. The data sources are the GTJA database and the Choice Financial Terminal. To eliminate dimensionality issues and improve model estimation accuracy, the natural logarithms of the two variables are taken. Additionally, this study does not examine holiday effects, and therefore the two datasets are treated as uniformly sampled discrete wavelet signals. Data analysis for this study is conducted using Matlab (R2023b).

**Fig 2:**
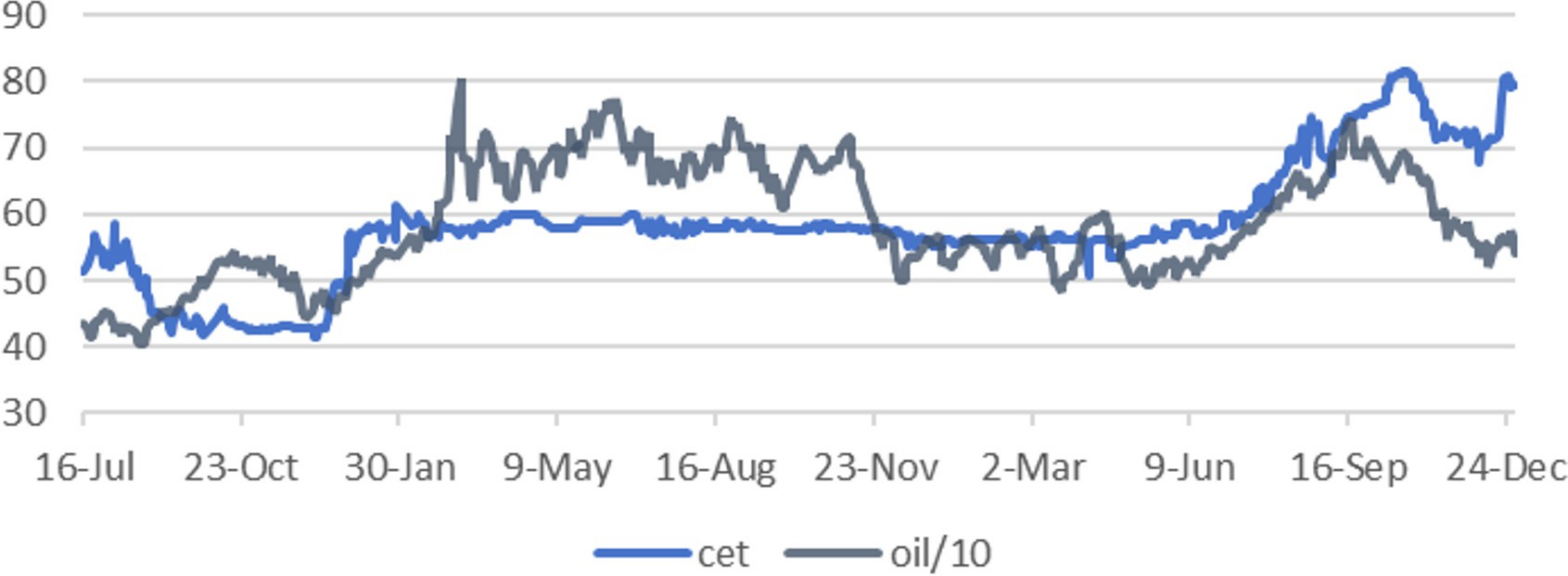
Price trends of China’s national carbon market and crude oil futures market.

As shown in Table 1, both the carbon market and crude oil futures exhibit right-skewed distributions, relatively small standard deviations, and concentrated data. Furthermore, the mean and median values are close, and there are no apparent outliers in the dataset. The correlation coefficient between the carbon market and the crude oil market is 0.53, indicating a positive correlation between the two markets. This is also corroborated in Fig 2, where it can be seen that, with the exception of the last quarter of 2021, the two markets tend to move in the same direction for most of the period. From Fig 2, it is also evident that the volatility of crude oil futures prices is larger compared to that of carbon prices.

**Table 1:** Descriptive statistics of the (daily) closing prices of the China’s national carbon market and crude oil futures market.

Row	Max	Min	Median	Mean	StdDev	Skewness	Kurtosis	R
lncet	4.40	3.72	4.06	4.05	0.15	-0.04	3.44	0.53
lnoil	4.39	3.70	4.05	4.06	0.15	-0.20	2.19	0.53

## 3. Empirical analyses

### 3.1 Parameter determination

In this paper, Daubechies Least Asymmetric wavelet filter is selected, where the length is 8, i.e. LA (8) wavelet filter. Because of its advantages, such as longer length and better phase, it is the main wavelet filter selected for MODWT of economic and financial time series in recent years. For example, Sui Xin [28] and Tang Yong [32] et al. also chose LA(8) wavelet in the exploration. The sample length of this paper is T = 596, L = 8 according to the following formula to determine the number of wavelet hierarchical level is 6.


$$J<log_{2}(T/(L‐1)+1)$$

The subsequent MODWT wavelet transform, the wavelet correlation leading lag is based on this wavelet basis function and decomposition scale expansion.

### 3.2 Empirical results

#### 3.2.1 MODWT

First, the ln(cet) and ln(oil) series are decomposed using MODWT into six levels, resulting in the D1, D2, D3, D4, D5, and D6 signals. These correspond to the detail coefficients for j = 1, 2, 3, 4, 5, and 6, respectively. The wavelet decomposition scales are then converted into time periods, with the D1 scale corresponding to a time period of 2–4 days, D2 corresponding to 4–8 days, D3 corresponding to 8–16 days, D4 corresponding to 16–32 days, D5 corresponding to 32–64 days, and D6 corresponding to 64–128 days. In this study, D1 and D2 are defined as short-term, D3 and D4 as medium-term, and D5 and D6 as long-term.

**Fig 3:**
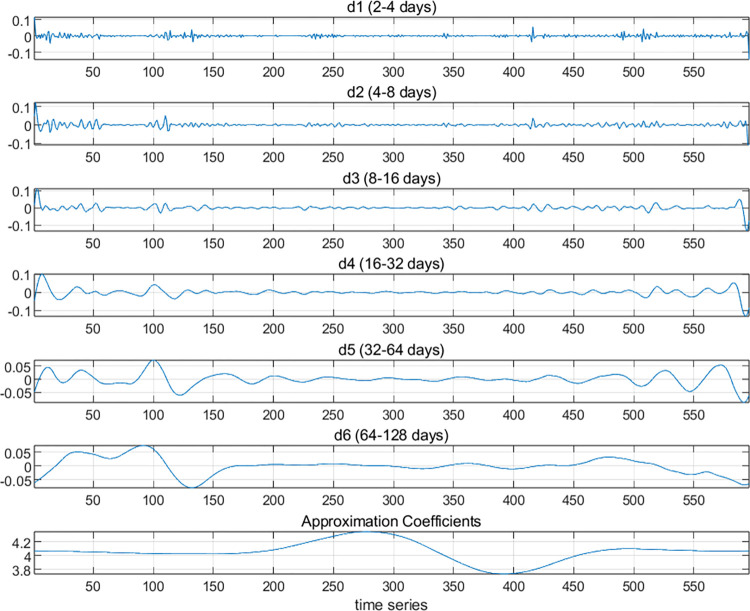
MODWT wavelet decomposition of China’s national carbon market.

**Fig 4:**
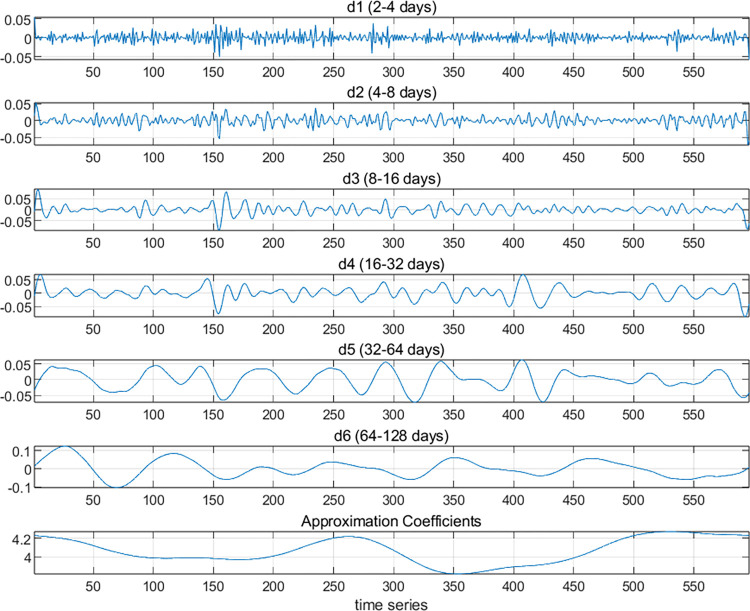
MODWT wavelet decomposition of crude oil futures market.

From the frequency domain perspective, the results of the MODWT wavelet decomposition are analyzed. As shown in Figs 3 and 4, both the carbon market and the oil market exhibit frequent short-term (D1 and D2) fluctuations, with oil futures prices fluctuating more frequently. This is because the oil futures market has a larger trading volume and more participants, leading to greater market activity and stronger liquidity. In the carbon market, the medium-term (D3 and D4) fluctuations are relatively mild, showing a pattern of sustained moderate volatility. The long-term (D5 and D6) fluctuations exhibit higher frequencies. In the oil market, both the medium-term and long-term fluctuations are more pronounced. This can also be confirmed from Fig 2. Overall, compared to carbon prices, oil futures prices are more volatile. From the time domain perspective, the carbon market experienced sharp fluctuations between time periods 80–120 (corresponding to mid-December 2021 to early January 2022) and 525–580 (corresponding to October and November 2023).

#### 3.2.2 Wavelet energy decomposition

The wavelet energy decomposition of carbon market and crude oil futures prices reveals the energy distribution of volatility at different scales. Wavelet energy decomposition involves breaking down a signal into components at different scales (frequencies) and then calculating the energy at each scale to identify which frequencies contribute most to the total energy. The results of the wavelet energy decomposition are shown in Table 2. For both the carbon market and the crude oil futures market, the energy is predominantly driven by long-term fluctuations. This indicates that both markets are primarily influenced by long-term volatility. In the case of the crude oil market, the dominance of long-term volatility is even more pronounced, with D6 (64–128 days) contributing more than 60%.

**Table 2:** Wavelet energy decomposition of China’s National Carbon Market and crude oil futures market.

	cet	oil
D1	0.0635	0.0287
D2	0.0751	0.0338
D3	0.1094	0.0938
D4	0.2086	0.1187
D5	0.2766	0.2107
D6	0.2669	0.5142

**Fig 5:**
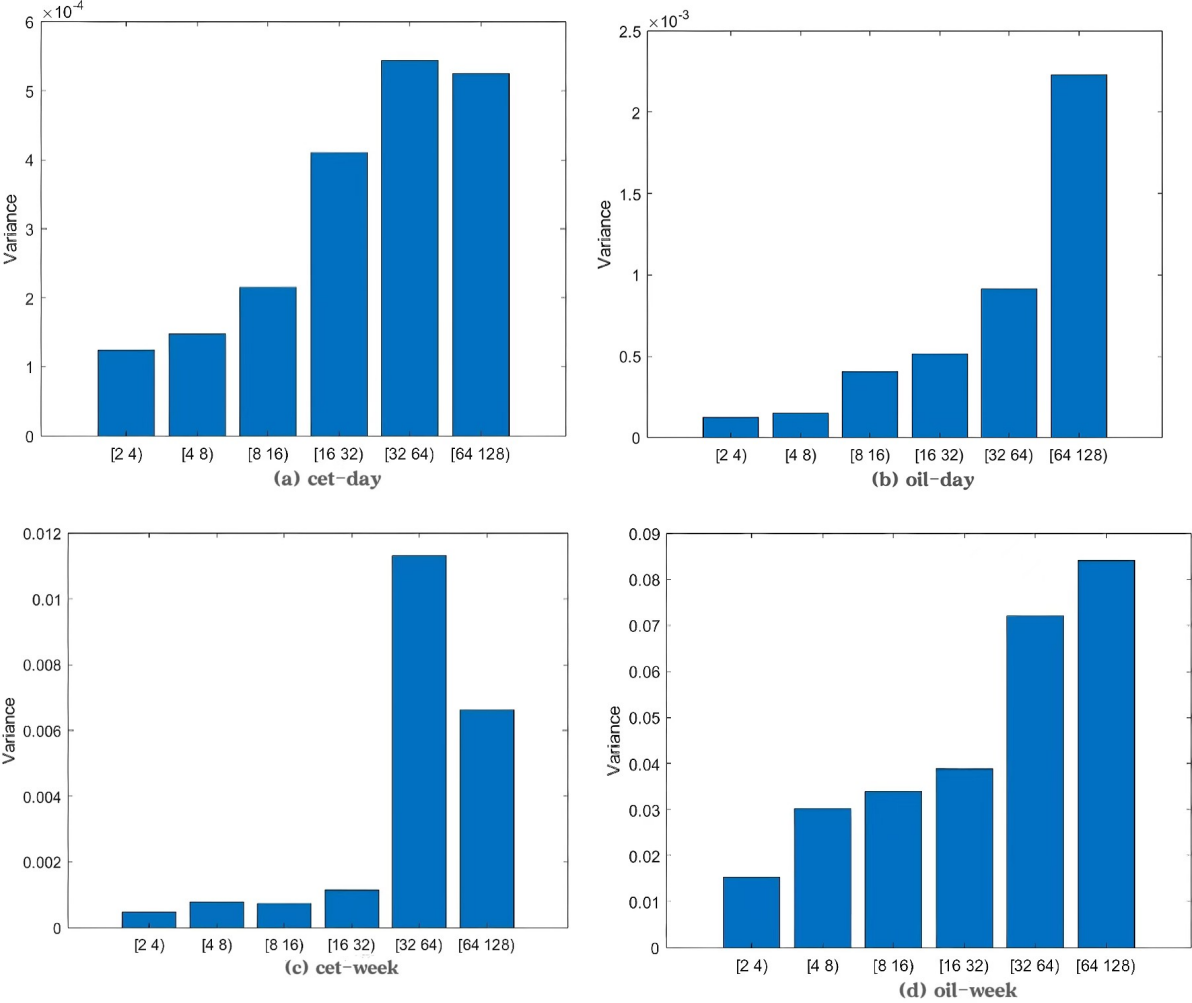
Variance plot of China’s national carbon market and crude oil futures market.

Variance is another statistical measure used to assess data distribution or volatility. By calculating the variance at different scales, we can also identify the contribution of each scale’s fluctuations to the overall volatility. As shown in Fig 5a and 5b present the variance of daily data for the carbon market and crude oil futures market, respectively. For both markets, the variance increases as the decomposition scale grows. Since daily data was used, decomposing into 6 levels does not yield extreme variance values at different scales. Therefore, we calculated the weekly average price from the daily closing prices and performed the decomposition again using weekly data. Fig 5c and 5d show the variance of the wavelet decomposition of weekly average prices for the carbon market and crude oil futures market, respectively.

In the carbon market, the variance is largest at the 32–64 (weeks) scale, indicating that long-term fluctuations dominate the volatility. In the crude oil futures market, the variance increases with the scale. The variance sharply increases at the 32–64 (weeks) scale, and remains higher at the 64–128 (weeks) scale than at the previous one. This suggests that the volatility in the crude oil market is also primarily driven by long-term fluctuations. Compared to the carbon market, the crude oil market shows a higher proportion of variance at higher scales, indicating that its long-term volatility is more intense.

In summary, both carbon prices and crude oil futures prices exhibit high-frequency fluctuations in the short term, with frequent price movements and no significant trends during this period. However, in the long term, both carbon prices and crude oil futures prices exhibit certain trend patterns. This aligns with the typical characteristics of financial markets, where short-term movements are often more random, while long-term trends are more influenced by fundamental factors, such as supply and demand, economic conditions, and policy changes. In conclusion, the carbon market, as part of the broader financial market, exhibits distinct characteristics typical of financial markets after analysis.

#### 3.2.3 Mean spillover

**Fig 6:**
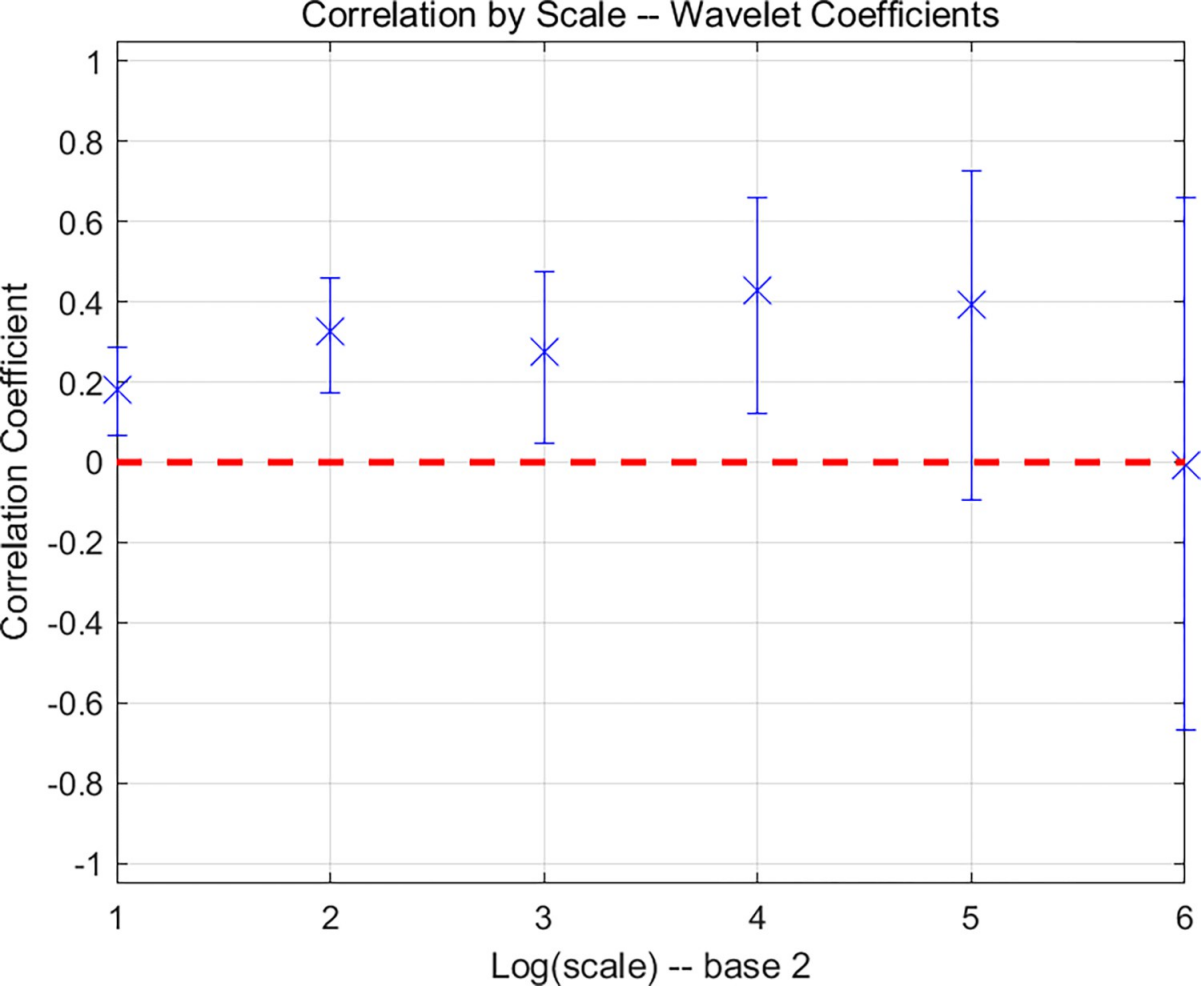
Graphical representation of wavelet correlation between China’s national carbon market and crude oil futures market.

This section analyzes the mean spillover between the two markets using wavelet coherence and lead-lag relationships. First, we discuss wavelet coherence. Wavelet coherence analysis is a method that utilizes wavelet transform to study the correlation between two time series, often employed to explore the correlation characteristics at different scales (frequencies) of the time series. In this paper, the MODWT wavelet transform is first applied to the carbon price and crude oil futures closing prices, followed by the calculation of wavelet coherence at different scales. The results are shown in Table 3 and Fig 6. The x-axis of Fig 6 represents the different decomposition scales, and the y-axis represents the correlation. This figure shows the correlation between the carbon market and the crude oil market at the 95% confidence interval. From the figure, it is evident that, at the D1–D4 scales, the two markets exhibit a significant positive correlation. As the decomposition scale increases, the width of the 95% confidence interval gradually increases, but it remains significantly greater than 0. At the D5 and D6 scales, the correlation is not significant. With the increase in the sample time period, the positive correlation between the two markets decreases. This indicates that in the short and medium term, the mean spillover between the carbon market and the crude oil futures market is significant, but it becomes insignificant in the long term.

**Table 3:** Wavelet correlation coefficients between China’s national carbon market and crude oil futures market.

	Lower	Rho	Upper	Pvalue
D1	0.07	0.18	0.29	0.00
D2	0.17	0.32	0.46	0.00
D3	0.05	0.27	0.47	0.02
D4	0.12	0.43	0.66	0.03
D5	-0.09	0.39	0.73	0.33
D6	-0.67	-0.01	0.66	0.37

**Fig 7:**
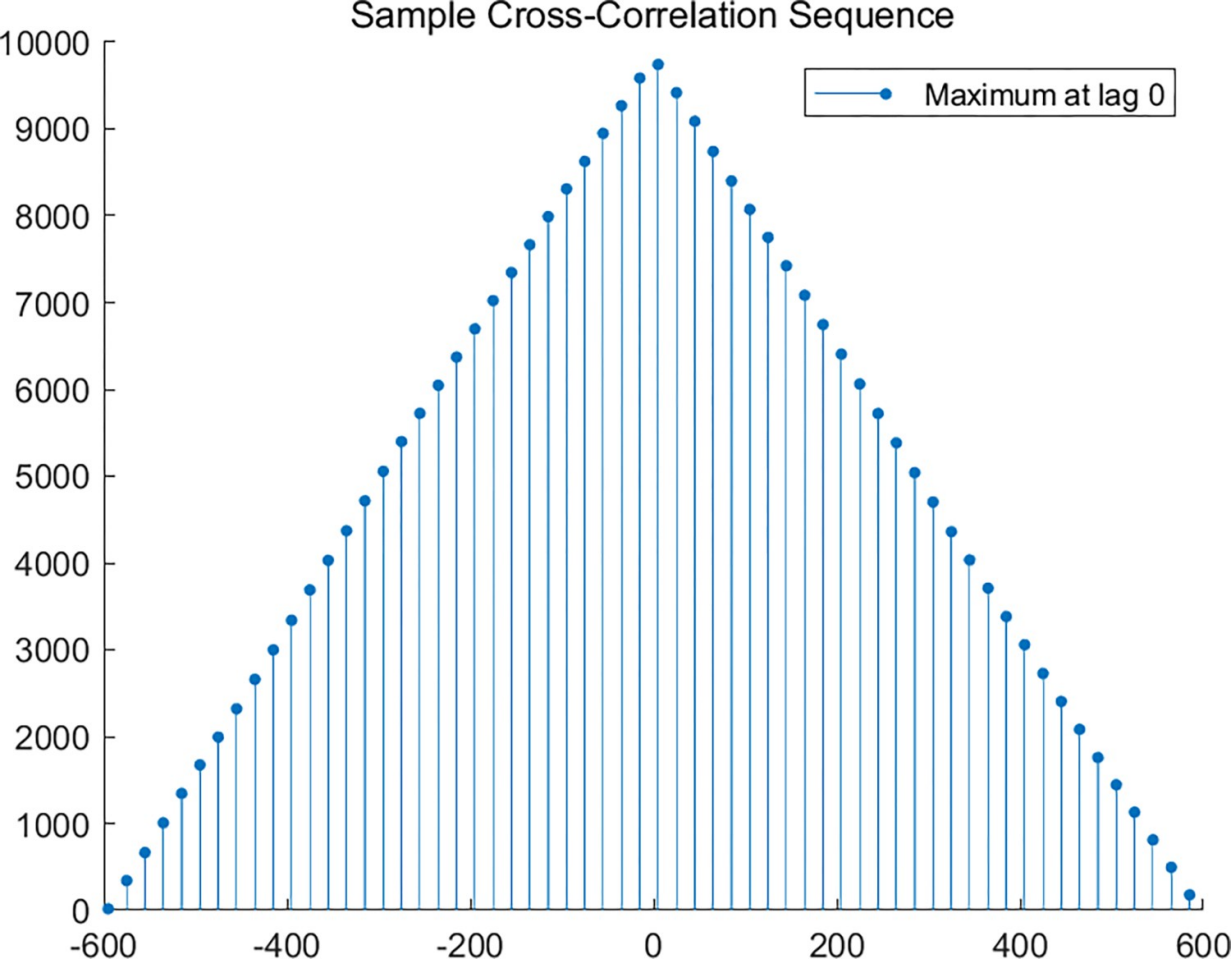
Lead-lag analysis between China’s national carbon market and crude oil futures market.

**Fig 8:**
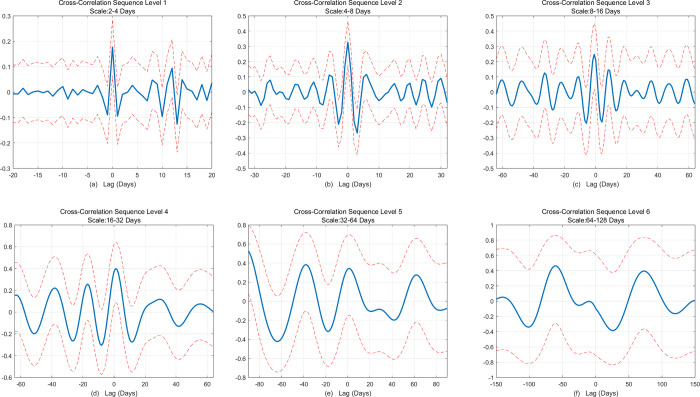
Analysis of different scales of lead-lag effects between China’s national carbon market and crude oil futures market.

Next, we then analyze whether there is a spillover effect between the carbon market and the crude oil futures market from the time-domain perspective. This paper uses the lead-lag relationship to investigate the connection between the two markets, aiming to determine whether one market can predict the other. The figure below first explores the overall relationship between the carbon market and the crude oil futures market. As shown in Fig 7, we calculate the correlation at different lag periods and determine the lag value corresponding to the maximum correlation coefficient. Based on this, we assess the existence of a lead-lag relationship between the two variables. From the figure, it can be seen that the correlation coefficient is highest when the lag period is 0. This suggests that, from an overall perspective, the changes in the carbon market and the crude oil futures market occur almost simultaneously, with no significant lag effect. Subsequently, we explore the lead-lag relationship between the carbon market and the crude oil market at different scales. Fig 8 consists of six subplots, which sequentially represent the lead-lag analysis between the two markets at the D1–D6 scales. The red line indicates the 95% confidence interval. By observing the first four subplots, it is evident that the carbon market and the crude oil futures market exhibit a significant positive correlation at lag 0. This means that, even at different scales, no lead-lag relationship exists between the two markets.

#### 3.2.4 Volatility spillover

**Fig 9:**
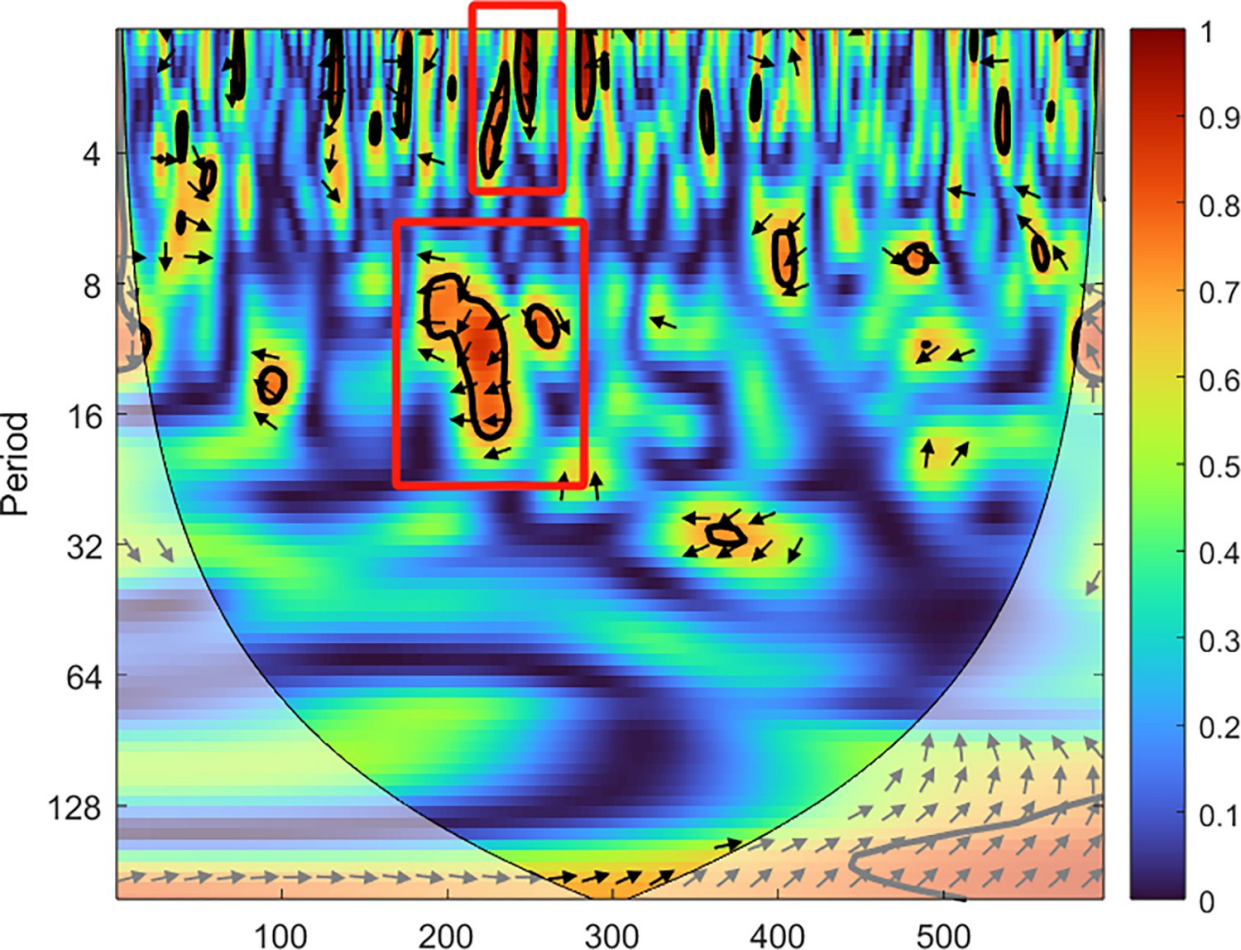
Wavelet coherence mapping of China’s national carbon market and crude oil futures market.

This paper uses wavelet coherence plots to investigate the volatility spillover effects between the carbon market and the crude oil futures market. Fig 9 shows the wavelet coherence structure of their interdependence. The legend on the right side represents the heatmap. The horizontal axis represents time, while the vertical axis represents frequency, which is shown as the duration of the study period (in days). Shorter periods correspond to shorter time scales or higher frequencies. The coherence magnitude is represented by the color of the frequencies. The redder the color, the greater the interdependence between the two series. Colder colors (blue) indicate weaker consistency. The black arrows in the coherence heatmap represent the causal relationship (lead-lag relationship) between the two time series. The direction of the arrow indicates the phase difference. Perfect correlation corresponds to zero phase difference, meaning no lead-lag relationship exists between the two markets. If the arrow points to the right (left), the correlation is positive (negative). If the arrow points upward (downward), the carbon market leads (lags) the crude oil futures market. The conical lines represent the valid boundary. When the arrow points diagonally, it indicates both interdependence and a lead-lag relationship. Therefore, the four types of arrows can be summarized as follows: (1) Right-upward arrows indicate that the carbon market leads the crude oil futures market with a positive correlation; (2) Right-downward arrows indicate that the crude oil futures market leads the carbon market with a positive correlation; (3) Left-upward arrows indicate that the carbon market leads the crude oil futures market with a negative correlation; (4) Left-downward arrows indicate that the crude oil futures market leads the carbon market with a negative correlation.

From Fig 9, we observe significant differences in the risk interdependence between the carbon market and the crude oil futures market at different times and frequencies. Specifically: First, at the D1–D2 scale, there are many areas enclosed by irregular thick black solid lines, which represent short periods but are distributed across different time intervals. This suggests that the risk interdependence between the carbon market and the crude oil futures market is strong in the short term. Second, at the D3–D4 scale, the number of areas enclosed by the thick black solid lines decreases significantly, and the distribution across different time periods is uneven. However, from the heatmap, the color of the areas enclosed by the thick black solid lines is more intense (red), indicating that the risk interdependence between the carbon market and the crude oil futures market significantly increases during specific moments in the medium term. Additionally, the lead-lag relationship between the two markets shows greater uncertainty. Third, at the D5–D6 scale, the risk interdependence between the carbon market and the crude oil futures market is low. Furthermore, Fig 9 reveals several highlighted areas with arrows pointing in different directions, further confirming the existence of volatility spillovers between the carbon market and the crude oil futures market. It also shows that the direction and intensity of this spillover effect are not fixed. In particular, during the period from 160 to 265 (March–August 2022), at the D1 to D2 time scales, the carbon market and the crude oil futures market exhibit significant volatility correlation, marked by downward arrows. This suggests that during this period, the crude oil futures market leads the carbon market. This period coincides with the initial outbreak of the Russia-Ukraine war, and the sharp rise in geopolitical risks led to a rapid increase in international oil prices, causing risk spillover from the crude oil futures market to the broader financial markets, including the carbon market. As time passed and the time scale shifted to D3 to D4, the volatility correlation between the carbon market and the crude oil futures market remained strong, but the relationship pattern changed. At this point, most of the arrows point leftward, revealing a dynamic inverse correlation, where the rise of one market is often accompanied by the decline of the other. This indicates that during this period, the risk in the crude oil futures market not only continued to spill over to the carbon market, but also the mutual influence between the two markets became more complex, presenting a scenario of mutual restraint. Additionally, this inverse relationship further confirms the existence of risk spillover between the carbon market and the crude oil futures market, and the direction and intensity of this spillover effect are not constant.

### 3.3 Discussion

First, we conduct a separate in-depth analysis of the carbon market and the crude oil futures market. Based on the empirical results presented earlier, we observe that both markets experienced significant volatility in the fourth quarter of 2021 and the fourth quarter of 2023. This volatility can largely be attributed to the specific behavioral patterns of market participants. Specifically, these two quarters coincided with the first and second compliance periods of China’s carbon market. This finding strongly supports the existence of a significant compliance-driven effect in China’s carbon market. In other words, around the compliance deadlines, when companies are required to settle carbon emission allowances, trading volumes surge, and price fluctuations intensify. In contrast, during non-compliance periods, trading volumes in the carbon market significantly decreases. This leads to reduced market activity and effectiveness. In stark contrast, the crude oil futures market exhibits frequent volatility throughout the observation period, indicating a higher level of market activity but also implying that investors face greater price fluctuation risks. From a volatility trend perspective, both the carbon market and the crude oil futures market are primarily influenced by long-term fluctuations. This suggests that in the short term, both carbon prices and crude oil futures prices may exhibit frequent and random fluctuations. However, from a long-term perspective, despite larger price fluctuations, there is still a discernible trend. This characteristic aligns with the general operating principles of financial markets, where short-term price movements are more random, while long-term trends are more likely to be influenced by fundamental factors such as supply and demand dynamics, the economic environment, and policy changes. In conclusion, the carbon market, as part of the broader financial market, does indeed exhibit clear financial market characteristics. However, its significant compliance-driven effect leads to lower market activity during non-compliance periods. This unique feature necessitates that policymakers and market participants pay close attention to and respond to the carbon market’s behavior, particularly in terms of price formation and risk management.

Next, we move to the analysis of mean spillover. As shown in the empirical analysis of the previous section, the carbon market and the crude oil futures market exhibit a significant positive correlation in the short and medium terms, and there is no leading-lag relationship between the two. This situation can be explained through the lens of supply-demand theory, externality theory, and market efficiency theory. From a supply-demand perspective, during periods of economic expansion, increased economic activity is typically associated with higher energy consumption. As crude oil is a key energy source, its combustion generates substantial carbon dioxide emissions, leading to an increase in overall carbon emissions. This in turn raises the demand for carbon emission allowances. The supply-demand effect drives up the carbon price. According to externality theory, carbon dioxide generated by crude oil combustion represents a negative externality. Its environmental damage is not reflected in the price of crude oil, and the rise in carbon allowance prices can be seen as a market adjustment to this negative externality. Ideally, carbon prices should signal to businesses that they need to reduce emissions, offering both constraints and incentives. For instance, as carbon prices rise, high-emission companies face increased pressure to cut emissions, while firms successfully transitioning to greener practices can sell excess carbon allowances for profit. This would lead businesses to reduce their reliance on fossil fuels, which in turn should lower energy market prices. In this ideal scenario, carbon prices and crude oil futures prices should exhibit an inverse relationship. However, the current relationship between the two in China is quite the opposite. This reflects the low effectiveness of the carbon market, as the price signals are not yet strong enough in the market. As a result, the incentives and constraints for emission-reducing companies remain weak.

Finally, we move to volatility spillover analysis. There is a clear volatility spillover relationship between the carbon market and the crude oil futures market, but this spillover relationship is not static. Overall, the correlation between the two markets is predominantly positive, and no leading-lag relationship exists. However, at certain times, temporary inverse correlations and instances where one market leads the other do occur. The good news is that this confirms the presence of significant risk spillover between these two markets, highlighting the risk contagion effect between financial markets. The bad news is that this also implies that investors face greater uncertainty when engaging in short-term investments. Different markets may react differently to sudden events, and short-term investors need to remain flexible and adapt based on the specifics of each situation.

## 4. Conclusions and recommendations

### 4.1 Conclusions

This paper uses methods such as MODWT wavelet transform, energy decomposition, wavelet correlation, lead-lag analysis, and wavelet coherence spectrum analysis to explore the volatility spillover effects between the carbon market and the crude oil futures market.

First, both the carbon market and the crude oil futures market exhibit frequent short-term fluctuations with significant characteristics of random walks. In the long term, however, there is a discernible trend. This indicates that, like the crude oil futures market, the carbon market possesses financial attributes. The difference, however, lies in the fact that the carbon market has a notable compliance-driven effect, with low activity levels during non-compliance periods. This conclusion provides important insights for the government in formulating relevant policies for both the carbon market and the crude oil futures market.

Second, in the time range from D1 to D4, both the carbon market and the crude oil futures market show a positive correlation, with no apparent lead-lag effects between them. Currently, the carbon market’s ability to constrain carbon emissions is not yet significant, meaning that the rise in carbon prices has not effectively and significantly curbed carbon emissions, and, consequently, has not had the expected impact on the crude oil futures market. This situation indirectly highlights the carbon market’s insufficient price discovery function, its relatively immature stage of development, and its low market efficiency. These factors offer critical reference points and directions for improvement as the government seeks to strengthen the carbon market and enhance its effectiveness.

Third, from both time-domain and frequency-domain perspectives, the carbon market and the crude oil market demonstrate strong volatility correlation, with this characteristic being particularly pronounced in the short- to medium-term. This finding reveals the inherent connection between the carbon market and the crude oil futures market, indicating that both markets share commonalities when facing other risk factors, which amplifies the potential risk of risk contagion. For short-term investors, this discovery offers important insights, suggesting that they should fully consider the interconnectedness and potential risks between the two markets when formulating investment strategies.

### 4.2 Recommendations

First, in the subsequent development of the carbon market, the primary task is to gradually expand the market trading volume. Efforts should also be made to develop market infrastructure in order to enhance market efficiency and ultimately achieve a significant increase in market effectiveness. Currently, China’s carbon market exhibits clear financial attributes, but its market liquidity is weak, and the price signaling function is ineffective. In future development, more emission-controlling companies should be gradually included to increase the carbon market’s trading volume. The government can attract more market participants by offering incentives such as tax breaks and financial subsidies. By increasing market activity, the price discovery function of the carbon market will be further strengthened, ultimately achieving a more effective reduction in carbon emissions through the carbon market.

Second, in terms of regulation, it is necessary for regulatory authorities to introduce appropriate regulatory frameworks and institutional rules, as well as to establish corresponding early warning measures. Improving the pricing mechanisms of both the carbon market and the crude oil futures market is crucial to reducing herd behavior between the two markets. It is also essential to minimize risk transmission between the crude oil and carbon markets, preventing excessive volatility in crude oil futures prices and carbon prices. Especially during extreme economic events, government departments should adopt necessary unconventional policy measures to prevent systemic financial risks. For example, increasing market transaction costs and guiding investor asset price expectations could help mitigate the impact of spillover effects.

Third, for emission-controlling companies, they should enhance their awareness of carbon asset management and improve their carbon asset management capabilities. They should also make full use of the correlation between the two markets to engage in rational investment hedging. Although the current national carbon trading market does not yet include carbon futures and forward contracts, preventing emission-controlling companies from directly implementing hedging strategies in the market, they can still cleverly utilize the correlation between the carbon market and the crude oil futures market to conduct cross-hedging. For example, when expecting carbon prices to rise, they can buy carbon allowances in the carbon market to lock in the current price while simultaneously selling crude oil futures contracts in the crude oil futures market. When expecting carbon allowance prices to fall, they can sell some carbon allowances in the carbon market to reduce inventory costs, while buying crude oil futures contracts to offset part of the loss from the drop in carbon allowance prices through profits from the crude oil futures contracts. The effectiveness of cross-hedging strategies should be regularly evaluated, including profitability, risk exposure, and changes in market correlations. It is also necessary for other crude oil futures investors to closely monitor fluctuations in the carbon market.

When exploring the risk spillover effects between the carbon market and the crude oil futures market, a major challenge faced by this paper is the relatively short start time of China’s national carbon trading market. This limits the amount of relevant data available for analysis, which may impact the comprehensiveness of the assessment of the risk spillover effects. Therefore, we plan to continue monitoring the development of China’s carbon market, and we expect to conduct further in-depth research and analysis by the end of 2025, using data with a longer time span and a more comprehensive dataset. This research aims to re-verify the findings of this study and will use rolling window techniques to further explore the dynamic evolution process of risk spillovers between the two markets.

## Supporting information

S1 Data (XLSX)
